# Gentisic acid sodium salt, a phenolic compound, is superior to norepinephrine in reversing cardiovascular collapse, hepatic mitochondrial dysfunction and lactic acidemia in *Pseudomonas aeruginosa* septic shock in dogs

**DOI:** 10.1186/s40635-016-0095-0

**Published:** 2016-07-26

**Authors:** Steven Mink, Subir K. Roy Chowdhury, Jose Gotes, Zhao-Qin Cheng, Krika Kasian, Paul Fernyhough

**Affiliations:** 1 Departments of Medicine and Pharmacology and Therapeutics, University of Manitoba, Winnipeg, MB Canada; 2 Division of Neurodegenerative Disorders at the St. Boniface Hospital Research Centre, Winnipeg, MB Canada; 3 Instituto Nacional de Ciencias Medicas y Nutricion Salvador Zubiran, Mexico City, Mexico; 4 Department of Medicine, University of Manitoba, Winnipeg, Manitoba Canada; 5 Department of Pharmacology and Therapeutics, University of Manitoba, Winnipeg, Manitoba Canada; 6 Health Sciences Centre, GF-221, 820 Sherbrook St, Winnipeg, MB R3A-1R9 Canada

**Keywords:** Lactic acidosis, ATP, Electron transport chain, Vasopressors, Antioxidants, Vasodilation, Infection, Septic shock, Mitochondria, Norepinephrine, Gentisic acid

## Abstract

**Background:**

The development of lactic acidemia (LA) in septic shock (SS) is associated with an ominous prognosis. We previously showed that the mechanism of LA in SS may relate to impaired hepatic uptake of lactate, but the mechanism was not clear. Uptake of lactate by the liver occurs by a membrane-associated, pH-dependent, antiport system known as the monocarboxylate transporter. In the hepatocyte, lactate can then be metabolized by oxidative phosphorylation or converted to glucose in the cytosol. In the present study, we examined (1) whether hepatic mitochondrial dysfunction accounted for decreased uptake of lactate in a canine model of *Pseudomonas aeruginosa* SS, (2) whether norepinephrine (NE) treatment by increasing mean arterial pressure (MAP) could improve mitochondrial dysfunction and LA in this model, and (3) whether gentisic acid sodium salt (GSS), a novel phenolic compound, was superior to NE in these effects.

**Methods:**

In anesthetized/ventilated dogs, we infused the bacteria over ~10 h and measured hemodynamics in various treatment groups (see below). We then euthanized the animal and isolated the hepatic mitochondria. We measured hepatic mitochondrial oxygen consumption rates using the novel Seahorse XF24 analyzer under conditions that included: basal respiration, after the addition of adenosine- diphosphate to produce coupled respiration, and after the addition of a protonophore to produce maximal respiration.

**Results:**

We found that in the septic control group, mean arterial pressure decreased over the course of the study, and that mitochondrial dysfunction developed in which there was a reduction in maximal respiration. Whereas both NE and GSS treatments reversed the reduction in mean arterial pressure and increased maximal respiration to similar extents in respective groups, only in the GSS group was there a reduction in LA.

**Conclusions:**

Hepatic mitochondrial dysfunction occurs in SS, but does not appear to be required for the development of LA in SS, since NE improved mitochondrial dysfunction without reversing LA. GSS, a phenolic compound restored mean arterial pressure, mitochondrial dysfunction, and LA in SS. This reduction in LA may be independent of its effect on improving hepatic mitochondrial function.

**Electronic supplementary material:**

The online version of this article (doi:10.1186/s40635-016-0095-0) contains supplementary material, which is available to authorized users.

## Background

The development of lactic acidemia (LA) in septic shock (SS) is associated with an ominous prognosis [1]. Blood concentrations of lactate depend on its rate of production and utilization by various organs [2]. Uptake of lactate by the liver occurs by means of a membrane-associated, pH-dependent, bidirectional facilitative antiport transport system known as the monocarboxylate transporter. Once transported into the hepatocyte, lactate can be converted into glucose by the gluconeogenic pathway or can be converted to pyruvate to enter the mitochondria to be metabolized to CO_2_ and water. The accumulation of lactate is usually associated with acidosis which may reflect ATP hydrolysis, extracellular transport of protons, among other mechanisms [1–8]. Hepatic mitochondrial dysfunction has been postulated as an explanation for the development of LA in SS, although extra-hepatic mechanisms also contribute [8–16]. In mitochondria, pyruvate undergoes conversion to acetyl CoA after which oxidative phosphorylation occurs. In this process, electrons are transferred primarily by reduced nicotinamide adenine dinucleotide (NADH) to mitochondrial complexes I, III, and IV to generate a proton (H^+^) gradient (the proton motive force; pmf) across the mitochondrial inner membrane. The free energy accumulated as the pmf is then used to drive ATP synthesis through the F1FoATP synthase activity (Complex V) allowing protons to return to the mitochondrial matrix [12, 13].

In the literature, the variable reports pertaining to hepatic mitochondrial dysfunction in SS may be a consequence of the different methodology and experimental protocols that were used in the different studies. The recent development of the Seahorse XF24 extracellular flux analyzer that we used in the present study allows for precise measurements of mitochondrial oxygen consumption under various conditions and has not previously been used to investigate hepatic mitochondrial dysfunction in SS (see Methods) [17].

Based on the numerous questions raised in the literature about the pathogenesis of LA in SS, we used a canine model in the present study to address the following questions. We first wanted to determine whether hepatic mitochondrial dysfunction evolved in this model and whether hepatic mitochondrial dysfunction could explain the development of LA that occurred. Secondly, we determined whether the finding of hepatic mitochondrial dysfunction in this model could be related to the accompanying systemic hypotension that develops in which we administered the vasopressor norepinephrine (NE) to reverse hypotension [18]. Thirdly, we previously showed that select phenolic compounds (see further below and Discussion) have unique physiological properties in our sepsis models, likely attributable to their antioxidant capabilities, in which they are able to reverse the systemic vasodilation and hence the low mean blood pressure (MAP) that develops in these models [19, 20]. In preliminary experiments, we found that gentisic acid sodium salt (GSS), could not only reverse the low MAP that occurs in our sepsis model, but could also decrease blood lactate concentrations. GSS contains two hydroxyl moieties attached to the benzoic acid ring (2,5-dihydroxybenzoic acid sodium salt) [21, 22]. In the present study, we compared the efficacy of GSS on systemic hemodynamics, LA, and hepatic mitochondrial function in our canine model with those of NE to determine whether GSS could be used as a new treatment of cardiovascular collapse and LA in SS.

## Methods

The University Animal Care Committee approved these canine experiments which conform with the Guide for the Care and Use of Laboratory Animals published by the US National Institutes of Health (NIH Publication No 85-23, 1996) [23]. The protocol reference number is 13-046/1/2 (AC 10865).

### Animal preparation

In this study, we used a bacteremic model of *Pseudomonas aeruginosa* as previously described [20] to measure hepatic mitochondrial function and LA under conditions in which there was an intense inflammatory response. In this protocol, we infused ~10^10^ colony forming units/h of *P. aeruginosa (ATCC 27853*) mixed in normal saline solution over the duration of the study, while in the non-septic groups, we administered normal saline solution as a placebo over a similar time frame (see groups below). Over the course of the experiment, we studied the animals (18 to 26 kg) under sufentanil citrate (0.05–0.3 ug/kg/min) and midazolam (5 μg/kg/min) anesthesia, in which the animals were ventilated while receiving 100 % oxygen to maintain arterial around PO_2_ ~ 400 mmHg. We increased the ventilator rate over the course of the study to attenuate the development of the metabolic acidosis that is observed in this model [20].

To measure hemodynamic parameters, we inserted two vascular catheters under sterile conditions as previously described [20]. We connected the catheters to respective pressure transducers (Cobe, Argon) that were referenced relative to the left atrium and that were connected to a chart recorder (Astro-Med, W Warwick, RI). To measure MAP, we inserted a polyethylene catheter into the right femoral artery, while to measure pulmonary vascular pressures, we inserted a Swanz-Ganz catheter (Edwards Lifesciences, Irvine, CA) by percutaneous techniques through the right jugular vein. From the Swanz-Ganz catheter, we obtained measurements of pulmonary arterial pressure (Ppa), wedge pressure (Pwp), and right atrial pressure (Rap), heart rate (HR) as well as determinations of thermodilution cardiac output (CO).

From the hemodynamic measurements, we calculated systemic vascular resistance (SVR) from [(MAP-Rap)/CO)*80]. We normalized measurements of CO and organ oxygen delivery (DO_2_) to body weight. We calculated oxygen delivery (DO_2_) from CO x Hb x 1.34 ml oxygen per gram of Hb, since Hb was 100 % saturated at a PO_2_ of ~400 mmHg. From the arterial catheter and distal port of the Swanz-Ganz catheter, we also took arterial and mixed venous blood samples, respectively, for analyses of PO_2,_ PCO_2_, and pH.

In addition, we placed another polyethylene catheter into the left jugular vein, also by percutaneous techniques, for infusion of the bacteria and intravenous fluids and for obtaining blood chemistry and hematological samples. Since the baseline Pwp found in this model usually averages ≈ 10 mmHg, we infused normal saline solution as necessary to maintain Pwp relatively constant over the duration of this study. Additionally, we obtained blood samples in each condition (see further below) that included monitoring of serum liver function tests, troponin, our index of myocardial damage, lactate concentrations, hematocrit (Hct) and WBC, among others. The Clinical Chemistry Laboratory at the Health Sciences Centre performed these clinical laboratory tests.

### Experimental protocols

We included five groups of animals in this study. These groups included a septic control group, a non-septic control group, a norepinephrine (NE) septic group, a gentisic acid sodium salt (GSS) septic group, and a GSS non-septic group. In each group, we made baseline measurements of hemodynamics and blood parameters after approximately 1-h period of stability. In the septic groups, after initiation of the bacterial infusion, we repeated the measurements in the septic shock condition. We defined the septic shock condition as the interval at which MAP decreased to 60–65 mmHg, since this would mark the MAP at which vasopressors would often be started in the clinical condition [18]. While the bacteria were still being administered, we started an infusion of NE in the NE septic group to return MAP to the ~ baseline value, while in an identical manner, we infused GSS in the gentisic septic group to maintain MAP comparable to the baseline value. We used the baseline value as the target MAP for NE and GSS to achieve, since this would be an easily identified end-point (see Discussion). In the septic control group, after the SS condition, we started an infusion of 5 % dextrose in water (D_5_W) at approximately 40 ml/h for the remainder of the study, since D_5_W was the diluent for both the NE and GSS treatments.

In the two non-septic groups, since the septic shock condition usually occurred ≈ 4.5 h after the start of bacterial infusion, we made measurements at comparable baseline and sham shock intervals in these groups. After the sham shock condition, we infused GSS mixed in D_5_W in the GSS non-septic group and infused D5W alone in the non-septic control group for the remainder of the study. In the GSS non septic group, we infused the GSS treatment at a comparable rate to that administered in the gentisic septic group.

In all groups, we then made post-treatment measurements at 3 and 5 h after completion of the septic shock/sham-shock determinations.

### Mitochondrial preparation

After completion of the in-vivo measurements, we administered sodium pentobarbital (110 mg/kg) to euthanize the animal. In a subset of animals of each group, we immediately harvested a piece of liver and isolated the mitochondria as described by Schnaitman and Greenawalt [24]. Briefly, we minced the extracted liver in mitochondrial isolation buffer (MSHE) that includes 70 mM sucrose, 210 mM mannitol, 5 mM : 4-(2-hydroxyethyl)-1-piperazineethanesulfonic acid (HEPES), 1 mM ethylene glycol tetraacetic acid (EGTA), and fatty acid free bovine serum albumin (BSA). We then disrupted the tissue using a drill-driven glass/Teflon dounce homogenizer. We centrifuged the homogenate three times, after which we re-suspended the final pellet in a minimal volume of MSHE + BSA and determined protein (mg/ml). We diluted the mitochondrial preparation in cold mitochondrial assay solution (MAS) that contained 70 mM sucrose, 220 mM mannitol, 10 mM KH_2_PO_4_, 5 mM MgCl_2_, 2 mM HEPES, 1 mM EGTA, and BSA for plating directly onto the custom Seahorse 24-well culture dish (~20 μg mitochondrial protein/50 μl per well; see further below). After centrifugation of the plate and addition of appropriate substrates, we viewed the mitochondria under a microscope to ensure consistent adherence to the well. We then transferred the plate to the XF24 Analyzer for initiation of the protocol as described below [17].

### Mitochondrial measurement of oxygen consumption rates (OCR)

To examine hepatic mitochondrial function in this model, we used a novel technology that is capable of measuring dynamic changes in mitochondrial function, not easily attainable by other technologies [17]. The Seahorse XF24 extracellular flux analyser (Seahorse Bioscience, MA, USA) uses a piston to reversibly enclose a small volume (7 μl) above the mitochondria that can monitor oxygen uptake in that volume for 2–5 min, then raises the piston, allowing the bulk incubation medium (~1 ml) to re-equilibrate. The ability to make up to four additions during the experiment allows mitochondrial respiration to be measured under various metabolic conditions. This piece of equipment has never been used to measure mitochondrial function in SS.

In this protocol, we sequentially measured mitochondrial function under various metabolic conditions [17]. To obtain basal OCR, we first added a substrate to the preparation that contained pyruvate (10 mM) and malate (2 mM). We then added ADP (adenosine- diphosphate; 4 mM) that allows ATP synthase to function, producing an increase in OCR (coupled OCR). When the ATP/ADP ratio approaches equilibrium, pmf rises, after which proton reentry through the synthase stops and respiration slows. We terminated coupled respiration by the addition of the ATP synthase inhibitor oligomycin (2.0 μM) where ATP recycling cannot contribute and there is a stable decrease in OCR. We then added the protonophore, FCCP (4 μM; carbonyl cyanide p-triflouromethoxy-phenylhydrazone). FCCP leads to uncoupled respiration in which we previously titrated the concentration of FCCP to yield a maximal effect. In mitochondria in which ATP synthase activity is a limiting factor, uncoupled respiration may be greater than coupled respiration. This indicates that the mitochondria have reached maximal respiration in uncoupled respiration in which this respiration is not limited by ATP synthase activity (i.e., ATP accumulation feedbacks on OCR inhibition). We finally added rotenone (2 μM; R) and antimycin A (2 μM; AA) which are inhibitors of the electron transport chain (ETC) Complexes, I and III, respectively. When added to the mitochondrial preparation, these inhibitors produce a marked decline in mitochondrial respiration since the electron transport chain is inhibited.

With each treatment group, we obtained measurements of basal OCR, coupled OCR, maximal OCR, and spare respiratory capacity from (uncoupled respiration minus basal OCR). We obtained two measurements during each condition in which we reported the average of the two measurements in the analysis.

### Determination of enzymatic activity of mitochondrial Complex IV (cytochrome *c* oxidase)

As part of our assessment of mitochondrial function, we also measured the enzymatic activity of hepatic mitochondrial Complex IV based on the rationale of Levy et al. [10] who considered that dysfunction of Complex IV was the most important determinant of abnormal mitochondrial function in SS. We determined Complex IV activity spectrophotometrically by a temperature-controlled Ultrospec 2100 ultraviolet-visible spectrophotometer (Biopharmacia Biotech, Uppsala, Sweden) [17]. Complex IV activity was measured at 25 °C by monitoring the absorbance decrease of reduced cytochrome *c* at 550 nm. The reaction was started by addition of 40 μmol/l reduced cytochrome *c* into 50 mmol/l phosphate buffer containing 2.5 μg mitochondrial protein solubilized with 0.02 % laurylmaltoside.

### Statistics

For the hemodynamic measurements in the 5 treatment groups, we used a two-way ANOVA (split-plot) in which we normalized hemodynamic data (CO) to body weight. In this statistical analysis, there were five groups of animals (i.e., five levels of factor A comparison) and four different time periods (four levels of B comparison, i.e., baseline, septic shock condition and 3 and 5 h posttreatment; GB-Stat V8, Dynamic Microsystems, Inc., Silver Springs, MD). From the results of the ANOVA tables, we used a Student Newman Keuls (SNK) multiple comparison test to determine where there were differences observed among the groups at the specific time periods. In other analyses, when there was only one level of the B comparison present, we used a one way randomized ANOVA and SNK. We used two tail tests with significance of *P* < 0.05. Results are reported as mean ± 1 SD.

## Results

Among the five treatment groups, the time to reach the septic condition from baseline was not different, measuring (mean ± SD) 4.1 ± 1.3 h (*n* = 13) in the septic control group, 4.9 ± 1.8 h (*n* = 10) in the norepinephrine septic group, and 4.8 ± 2.4 h (*n* = 11) in the gentisic septic group. In the non-septic control group (*n* = 16) and the GSS non septic group (*n* = 5), we made measurements at 4.5 h post baseline in the sham-shock condition. In the five groups, there were no differences in the mean animal body weights, measuring 20.3 ± 2.7 kgs in the septic control group, 20.7 ± 1.7 kgs in the NE septic group, 22.3 ± 2.4 kgs in the gentisic septic group, 21.1 ± 2.7 kgs in the non-septic control group, and 23.6 ± 1.9 kgs in the GSS control group.

There were also no differences in the total amount of volume infused in the three septic groups over the course of the experiment, measuring 6 ± 1 l in the septic control group, 6 ± 1.6 l in the NE septic group, and 6.2 ± 1.7 l in the gentisic septic group. On the other hand, we administered significantly less fluid in the non-septic groups (*P* < .05) which measured 3.7 ± 1 l in the non septic and 3.4 ± 0.6 l in the GSS non septic group. At the time of autopsy, there was a large amount of ascites found in the septic groups which was not found in the non-septic groups and which accounted for the large positive fluid balance observed in the septic groups (see Discussion). All animals in each of the five groups survived the last measurement condition.

### Hemodynamic measurements and laboratory results

**Fig. 1:**
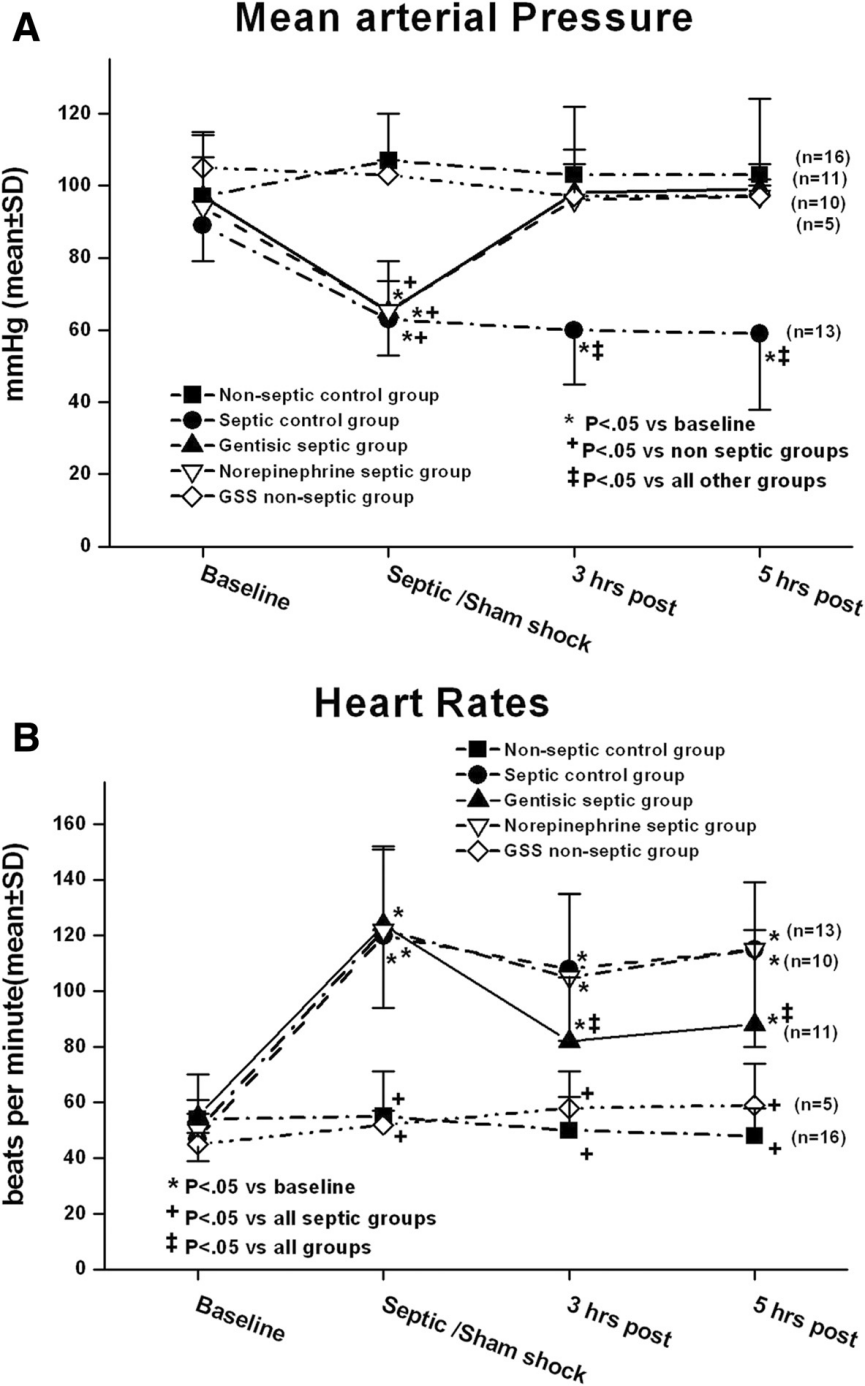
In panel **a** in the three septic groups, mean arterial pressure (MAP) in the septic shock condition decreased to approximately 60 % of baseline. In the gentisic septic group and the norepinephrine (NE) septic group, both treatments increased MAP back to the baseline value. In panel **b** heart rates increased in the septic groups over the course of the study. In the gentisic septic group, heart rates were lower at 3 and 5 h post-treatment conditions as compared with the other septic groups. GSS is gentisic acid sodium salt. Statistical analyses included two-way repeated measures analysis of variance and Student Newman Keuls (SNK) multiple comparison multiple comparison test

At baseline, there were no differences in MAP among the five groups which averaged around 100 mmHg (see Fig. 1a). In the two non-septic groups, MAP remained at this baseline value for the remainder of the study, while in three septic groups, mean MAP decreased to approximately 60 mmHg at the septic shock condition. In the septic control group, MAP remained at this low value for the duration of the experiment. On the other hand, in both the NE and gentisic septic groups, treatments caused a return in MAP to baseline where MAP remained for the 3 h post- and 5 h post-treatment intervals. The increases observed with both treatments occurred within minutes of infusion. There was no change in MAP observed in the non-septic GSS group which is identical to that observed in other studies when similar phenolic compounds were infused in non-septic animals [19].

The average dose of NE infused over the 5-h interval was 0.51 ± 0.22 μg/kg/min. In the gentisic septic group, the average dose of GSS infused over the same interval was 0.55 ± .29 mg/kg/min. The average infusion rate of GSS in the GSS non-septic group was 0.48 ± .008 mg/kg/min.

In the septic shock condition, the mechanism for the reduction in MAP among the septic groups was due to a decrease in SVR which decreased to similar extents among the three groups (see Table 1). Both GSS and NE treatments tended to increase SVR at 3 and 5 h post-treatment as compared with the septic control group, but these results did not reach statistical significance among the groups. In the GSS non-septic group and the non septic control group, there were no significant changes in SVR with treatment.

**Table 1:** Systemic vascular resistances in the five groups (dynes S/cm^5^: mean ± SD)

	Baseline	Septic/sham shock/condition	3 h post	5 h post
Non-septic control group (n = 16)	2299 ± 603	2794 ± 1024+	2819 ± 1114	2829 ± 1007
Septic control group (*n* = 13)	2583 ± 803	1351 ± 374*	1745 ± 1037	2226 ± 1503
Gentisic septic group (*n* = 11)	2717 ± 605	1272 ± 695*	2767 ± 607	3234 ± 1013#
Norepinephrine septic group (*n* = 10)	2778 ± 526	1539 ± .375*	2573 ± 968	2767 ± 1063
Gentisic non-septic group (*n* = 5)	2740 ± 340	2416 ± 499	1798 ± 256	1866 ± 25

Statistics by two way analysis of variance (ANOVA) and Student Newman Keuls multiple comparison test that included the five groups and four time periods. *P < 0.05 vs baseline; +*P* < 0.05 vs all septic groups; #*P* < 0.05 vs gentisic non septic group

For the most part, CO remained relatively unchanged over the course of the study in the five groups (see Table 2). In the gentisic septic group, although CO increased in the septic shock condition prior to GSS treatment, by the end of the study, CO were similar among the groups.

**Table 2:** Cardiac indices in the five groups (l/min/kg: mean ± SD)

	Baseline	Septic/sham shock/condition	3 h post	5 h post
Non-septic control group (*n* = 16)	0.15 ± .03	0.15 ± .05	0.14 ± .04	0.13 ± .03
Septic control group (*n* = 13)	0.13 ± .04	0.17 ± .04	0.15 ± .07	0.11 ± .06
Gentisic septic group (*n* = 11)	0.12 ± .02	0.20 ± .08*+	0.12 ± .03	0.11 ± .04
Norepinephrine septic group (*n* = 10)	0.12 ± .02	0.15 ± .03	0.14 ± .03	0.14 ± .04
Gentisic non-septic group (*n* = 5)	0.12 ± .02	0.13 ± .02	0.16 ± .04	0.16 ± .05

Statistics by two way analysis of variance (ANOVA) and Student Newman Keuls multiple comparison test that included the five groups and four time periods. *P < 0.05 vs baseline; +*P* < 0.05 vs non septic control group

In addition, we found that HR increased in the three septic groups during bacterial infusion from a mean of about 60 bpm at baseline to about 120 bpm at the septic shock condition (Fig. 1b). In the gentisic septic group, this treatment caused a significant decrease in HR at 3 and 5 h post-treatment as compared with the other septic groups. As well, we found that troponin, our index of myocardial injury, increased in the NE septic group at the 5 h interval, while we did not observe this finding in the other septic groups (see Additional file 1: Figure S1).

Although, in this protocol, we attempted to keep Pwp close to the baseline value, there were slight changes in Pwp observed over the course of the study in the five groups, particularly in the non-septic groups, since we needed to constantly infuse intravenous volume to administer the anesthetic agents in the latter groups. Overall, however, these changes were small (see Table 3) (see Discussion).

**Table 3:** Left ventricular filling pressures (Pwp) in the five groups (mmHg: mean ± SD)

	Baseline	Septic/sham shock/condition	3 h post	5 h post
Non-septic control group (*n* = 16)	10.8 ± .1.6	12.8 ± 1.8*%	13.8 ± 2.1*%	13.9 ± 1.8!
Septic control group (*n* = 13)	10.7 ± 1.1	8.8 ± 1.6*	9.4 ± 1.4#	9.3 ± .1.4#
Gentisic septic group (*n* = 11)	10.2 ± 1.6	9.3 ± 2.6	11 ± 2.6	12 ± .2.9*
Norepinephrine septic group (*n* = 10)	10.0 ± .2	9.0 ± 1.6	10.2 ± 2.8	10.1 ± 2.7#
Gentisic non-septic group (*n* = 5)	13.0 ± 2.6+	14.2 ± 3.7 %	14.2 ± 2.2 %	13.6 ± 4.2 %*

Statistics by two way analysis of variance (ANOVA) and Student Newman Keuls multiple comparison test that included the five groups and four time periods. *P < 0.05 vs baseline; %*P* < 0.05 vs all septic groups; #*P* < 0.05 vs gentisic septic group; !*P* < 0.05 vs septic control group and NE (norepinephrine) septic group; +*P* < 0.05 vs other groups

**Fig. 2:**
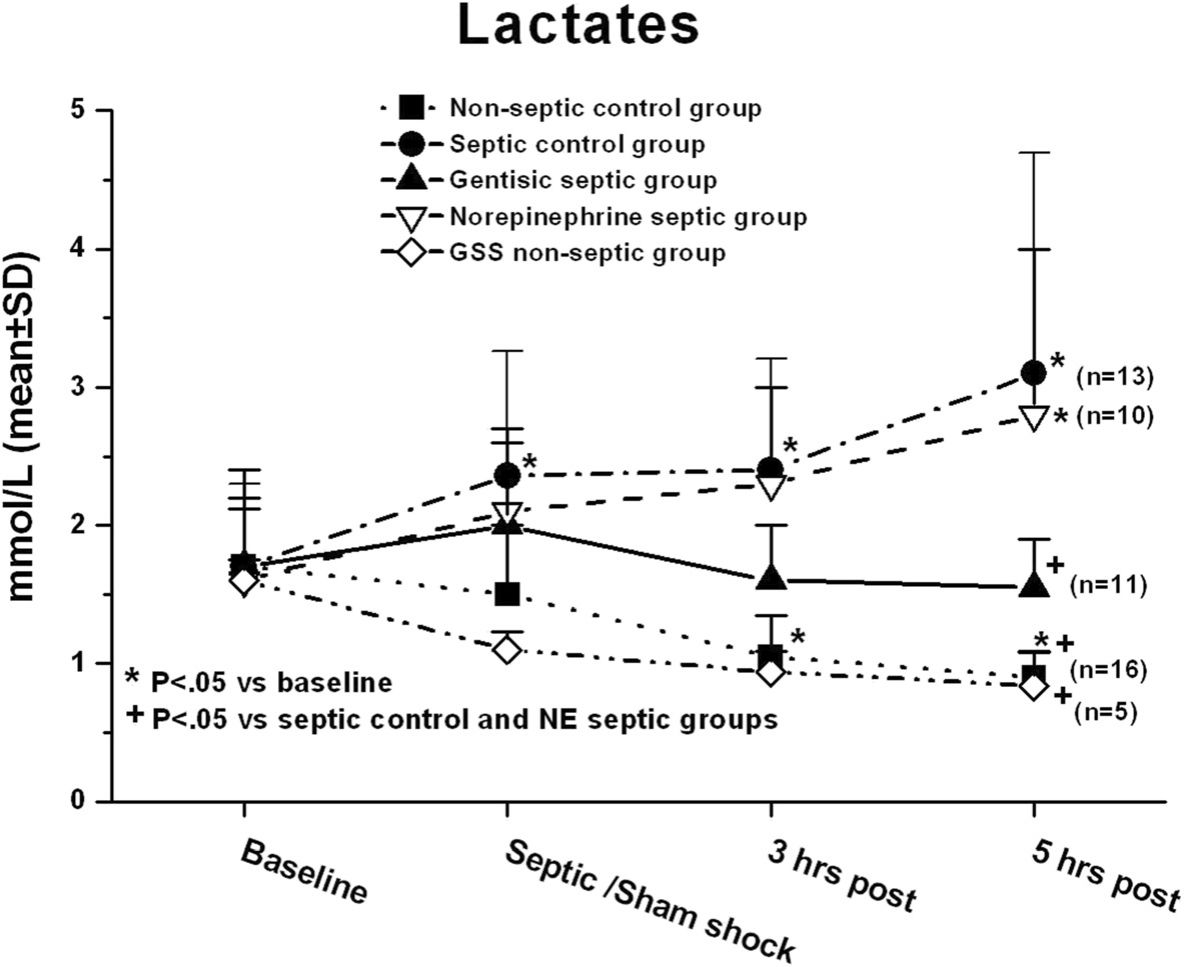
Serum lactate increased in the NE septic group and the septic control group over the course of study, while it decreased in the non septic groups and did not change in the gentisic septic group. GSS is gentisic acid sodium salt. Statistical analyses included two-way repeated measures analysis of variance and Student Newman Keuls (SNK) multiple comparison multiple comparison test

The changes in blood lactate differed among the five groups (Fig. 2). In the two non-septic groups, there was a progressive decline in blood lactate concentrations over the course of the study. On the other hand, blood lactate concentrations significantly increased in the septic control group and the NE septic group toward the end of the study, while blood lactate concentrations did not increase and even tended to decrease slightly in the gentistic septic group.

To determine the extent to which oxygen delivery might have affected the changes in lactate concentrations with the different treatments, we also calculated DO_2_ in the five groups (see Additional file 2: Table S1). We found that on the mean DO_2_ at the end of the study was lowest in the septic control group. In the NE septic group, NE treatment caused an increase in DO_2_ as compared with the septic control group and the gentisic septic group. In terms of the latter effect, this was most due to an increase in hemoglobin, since CO did not change (see Additional file 3: Table S2, Additional file 4: Table S3, Additional file 5: Table S4, Additional file 6: Table S5 and Additional file 7: Table S6). Ph at the end of the study was higher in the gentisic septic group, while mixed venous PO_2_ were not different among the groups. Changes in other laboratory parameters among the groups were small (see Additional file 3: Table S2, Additional file 4: Table S3, Additional file 5: Table S4, Additional file 6: Table S5 and Additional file 7: Table S6; see Discussion).

### Mitochondrial measurements

**Fig. 3:**
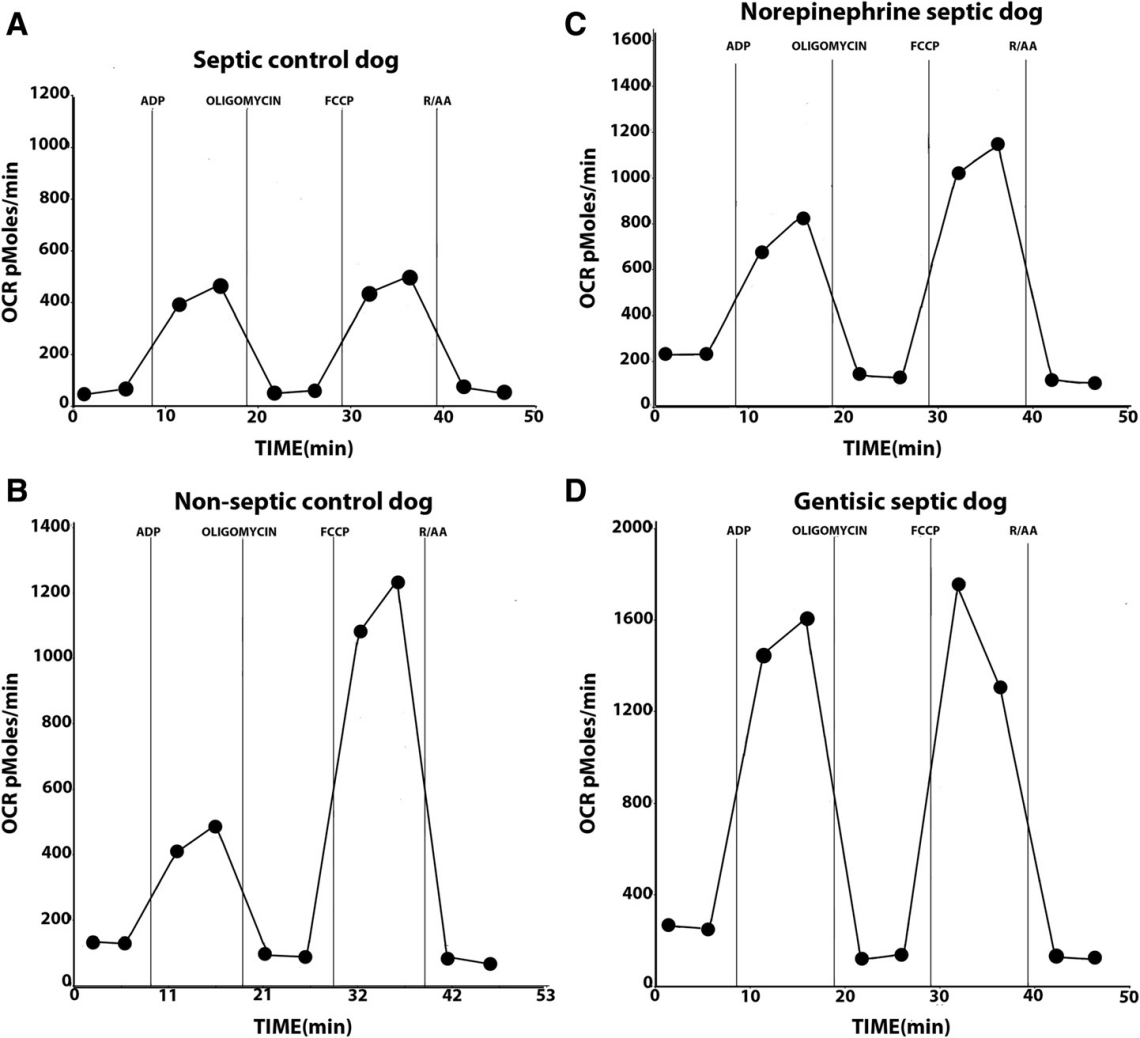
Oxygen consumption rates (OCR) were measured in the Seahorse Instrument and examples are shown from mitochondria harvested from an animal in the septic control group (panel **a**) in a non-septic control dog (panel **b**) in the norepinephrine (NE) septic group (panel **c**) and in the gentisic septic group (panel **d**) under different metabolic conditions. In each experiment, we obtained two measurements in each condition and the average of the two conditions was obtained. In panel **a** basal OCR was slightly low in the animal in the septic control group as compared with the non septic control group. When adenosine diphosphate (ADP) was added to the preparation, OCR rate in the septic animal in panel A was not different from the animal in the non-septic control group (panel **b**). However, the response to FCCP (carbonyl cyanide p-triflouromethoxy-phenylhydrazone) in the animal in the septic control group (panel **a**) was attenuated as compared with the animal in the non-septic control group (panel **b**). With norepinephrine treatment, there was a restoration in the response to FCCP in the NE septic group (panel **c**). In the animal in the gentisic septic group, there was a large response in OCR to ADP which nearly equaled the response to FCCP (panel **d**)

In the mitochondrial preparation, the predominant effect of SS on oxygen consumption rates (OCR) was to cause a decrease in maximal respiration. Examples of OCR under the different mitochondrial conditions for the different groups are shown in Fig. 3. In the septic control group (Fig. 3a), basal OCR was slightly less than values found in the other groups. When we administered ADP to the preparation, OCR in the septic control group increased to an extent not much different than that found in the non-septic control group (Fig. 3b). In contrast, when we added the protonophore FCCP at 4 μM to the preparation, there was a large increase in OCR in the non-septic control group (Fig. 3b) that was attenuated in the septic control group (Fig. 3a). In the NE septic group, the predominant effect of NE treatment was to restore the increase in OCR in response to FCCP (Fig. 3c), such that the effect of FCCP on OCR was now similar to that found in the non-septic control group. In the gentisic septic group (Fig. 3d), in addition to restoring basal respiration to the non-septic control value, GSS treatment produced a greater increase in coupled respiration as compared to the other groups. In this case, the OCR response with ADP was quite similar to that found with FCCP of 4 μM. In the GSS non-septic group (data not shown in this figure), the results were very similar to those found in the non-septic control group (see further below).

**Fig. 4:**
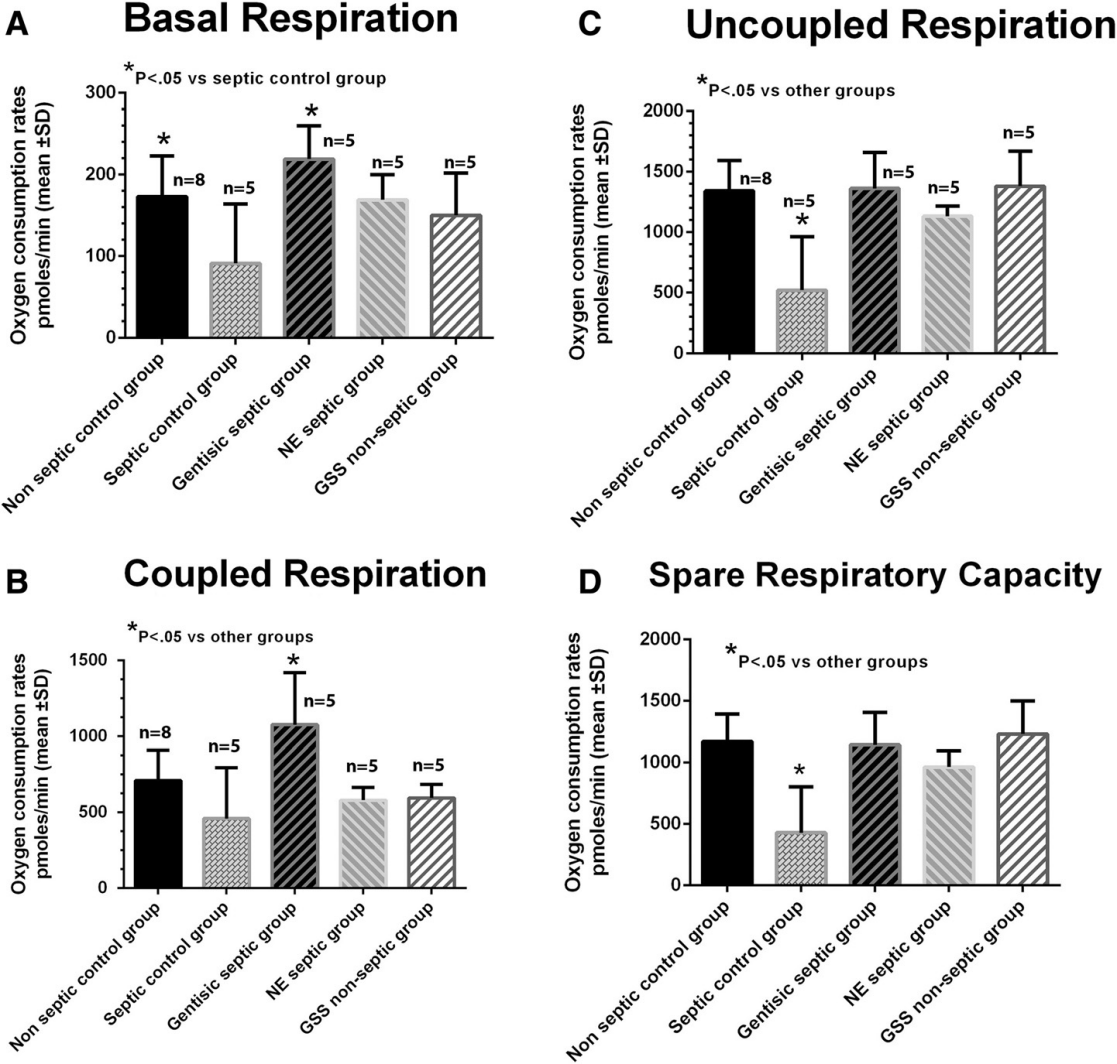
Mean oxygen consumption rates (OCR) under the different metabolic conditions are shown for the five groups. In panel **a** basal OCR in the septic control group was significantly less than that measured in the gentisic septic group. In panel **b** coupled respiration in the gentisic septic group was significantly greater than the other groups. In panel **c** uncoupled (maximal) OCR in the septic control group decreased as compared with the other groups in which we used a FCCP of 4 μM (i.e., maximal) in the analysis. In panel **d** spare respiratory capacity was decreased in the septic control group as compared with the other groups. Spare respiratory capacity was calculated from the difference between uncoupled and basal respiration (see text for Discussion). Statistical analyses included a one way analysis of variance and Student Newman Keuls (SNK) multiple comparison multiple comparison test

The mean results of mitochondrial OCR parameters are shown in Fig. 4. In Fig. 4a, there was a decrease in basal respiration in the septic control group, particularly as compared with that found in the gentisic septic group, while coupled respiration in the septic control group was not different from that found in the non septic control group (4B). However, in the gentisic septic group, coupled respiration was significantly higher than in the other groups. The results obtained when we added FCCP at 4 μM to the preparation to achieve maximal (uncoupled) respiration are shown in Fig. 4c. In the septic control group, uncoupled respiration was decreased as compared with the other groups, while there was no difference in maximal OCR among the other groups. In addition, spare respiratory capacity (see Fig. 4d), calculated as the difference between maximal and basal respiration, was significantly reduced in the septic control group as compared with the other groups.

In addition, enzymatic activity of mitochondrial Complex IV significantly decreased in the septic control group (2.87 ± .73 μmol/min/mg protein) as compared with the non septic control group (4.55 ± .98) (*P* < .01), while the values in the gentisic septic group, NE septic group, and GSS non septic group were 3.68 ± .59, 3.89 ± .33, 3.01 ± .66, and 2.44 ± .63, respectively. These results were not different among the groups (see Discussion).

## Discussion

We designed this study to address several questions. One objective was to determine whether hepatic mitochondrial dysfunction developed in this model of SS. To this effect, we showed that after approximately 10 h of bacteremia in this model, there was evidence that mitochondrial dysfunction had evolved. There was a significant reduction in maximal respiration found in the septic control group, while basal respiration and mitochondrial Complex IV enzymatic activity also appeared depressed. Coupled respiration did not decline. In the literature, the question of whether mitochondrial dysfunction develops in SS has been debated [16, 25, 26]. Although the explanation for differences in the literature is not clear and may be a consequence of the diverse models and methodologies used, most of the previous studies have concentrated either on coupled respiration (often termed State III) or the ratio of State III to State IV (in which oligomycin is added to the preparation) as their primary index of mitochondrial function. In contrast, our results demonstrate that uncoupled, maximal respiration may be a more sensitive parameter to reflect mitochondrial dysfunction in sepsis. In SS, because of the high metabolic energy demands that occur, hepatic mitochondrial ATP may be utilized as quickly as it is formed. Since there would be no feedback of ATP on inhibiting ATP synthase activity, uncoupled maximal respiration may be a more sensitive parameter of the mitochondrial dysfunction that occurs in patients in septic shock.

Another objective of this study was to determine whether NE treatment could reverse hepatic mitochondrial dysfunction in septic shock, since NE is often administered to increase blood pressure in the clinical condition. We found that in the NE septic group, NE resulted in an augmentation of maximal OCR to an extent comparable to that observed in the non-septic control group. In the literature, the effect of NE on mitochondrial function in sepsis has been controversial. Regueira and colleagues [27] found that in an endotoxemic pig model, administration of NE was associated with an improvement of hepatic mitochondrial respiration and concluded that this effect was probably mediated by a direct effect of NE on liver cells. On the other hand, in a fecal model of peritonitis in pigs, Vuda, and colleagues [25] did not find that NE improved mitochondrial function when they measured the in vitro effect of NE on respiratory efficiency of Complex I and Complex II of hepatic cells taken after prolonged exposure to sepsis.

NE treatment could prevent mitochondrial dysfunction by increasing hepatic oxygen delivery, thereby preventing oxidant damage produced by ischemic hypoxia, or by an intrinsic effect. NE has been shown to cause an increase in calcium transport into the mitochondrial matrix [28]. Calcium can stimulate three different dehydrogenases of the citric acid cycle increasing the substrate availability of NADH to the respiratory chain that could lead to an increase in mitochondrial respiration. Although the present study does not elucidate the mechanism involved, we found that NE treatment resulted in an increase in DO_2_ in the NE septic group as compared with the septic control group (see Additional file 2: Table S1). In a previous study [3], we found that hepatic blood flow was well-maintained in a NE treated septic group as compared to an untreated septic group [3]. Thus, an increase in oxygen delivery to the liver could have reversed any contribution of ischemic hypoxia to the mitochondria and prevented mitochondrial oxidant damage in the NE treated septic group [7–9]. Although this study does not delineate the mechanism involved, the fact that NE can reverse mitochondrial dysfunction in SS provides a rationale for its use in the treatment in the clinical situation.

Despite the fact that NE reversed hepatic mitochondrial dysfunction in the NE septic group, our results importantly show that this effect did not lead to an attenuation of LA. Thus, hepatic mitochondrial dysfunction is not necessary for the development of LA in SS. In the NE septic group, we observed that mean blood lactate concentration increased over the course of the study, such that at the 5-h measurement period, serum lactate was not different from that observed in the septic control group. In a previous study, we did not find that NE infusion altered hepatic lactate extraction, hepatic blood flow, or splanchnic lactate production in a similar animal model [3], although it is possible that the high lactate concentration found in the NE septic group in the present study was related to increased lactate production from extra-hepatic sources, such as skeletal muscle [29]. Nevertheless, although there may be a variety of extra hepatic sources of lactate that contribute in sepsis under various conditions, we previously observed that the canine liver could efficiently metabolize very large quantities of lactate during lactate loading under non septic conditions [3]. One would expect therefore that this very large reserve capacity of the liver would be able to overcome any contribution of lactate from an extra-hepatic source maintaining lactate concentrations low. Taken together, these findings suggest that hepatic impairment of lactate metabolism in SS may also reflect a non-mitochondrial mechanism, and that LA may occur in SS despite the finding of normal hepatic mitochondrial function. Whereas it is likely that hepatic mitochondrial dysfunction contributes to LA in SS under conditions when MAP decreases to a value low enough to cause severe mitochondrial hypoxia, we believe that an impairment of hepatic metabolism of lactate may also occur through other mechanisms in this condition, such as those that involve accelerated glycolysis [30] or impaired gluconeogenesis [31] (see further below).

In contrast to NE treatment, GSS was capable of improving the three most important parameters measured in this study, namely MAP, hepatic mitochondrial dysfunction and LA. GSS contains two hydroxyl moieties attached to the benzoic acid ring (2,5-dihydroxybenzoic acid sodium salt) and is a breakdown product of aspirin [21, 22]. GSS has potent antioxidant properties that might account for its greater beneficial effect than NE on mitochondrial function in this model. In the present study, not only did GSS result in an increase in maximal OCR as compared with the septic control group, but GSS caused a marked increase in coupled respiration and an increase in basal respiration. Whereas there was only a trend for GSS to increase mitochondrial Complex IV by enzymatic measurements, we think that measurements of OCR by the Seahorse Instrument provide a much more sensitive indication of mitochondrial function than does the assessment of mitochondrial enzymatic activity of the different complexes. The electron transport chain comprises multiple enzymatic complexes of which one or all may be compromised in SS. Brealey and colleagues [32] found that Complex IV activity was preserved in a long-term rodent model of sepsis, while activity of Complex I decreased. We measured Complex IV activity in this model based on the work of Levy et al. [10] and did not measure Complex I activity. Different results in the literature may reflect the various preparations and models that were used. However, an advantage of the Seahorse Instrument is that it gives an overall functional assessment of the entire electron transport chain in which the functionality of whole chain is assessed, while the significance of small reductions in enzymatic activity of the different complexes in terms of their role in reducing OCR may be difficult to assess.

In the gentisic septic group, the mechanism by which GSS enhanced mitochondrial function was most likely related to GSS’s antioxidant effects that could lead to an increase in coupled respiration by enhanced transfer of ATP out of the cell by adenine nucleotide translocase. This could lead to less feedback inhibition of respiration by ATP, such that mitochondrial coupled respiration would be higher in the gentisic septic group. It is also important to note that not all phenolic compounds have the capacity to reduce LA in this SS model, since we did not find this effect to occur when we administered ethyl gallate in a similar manner [19]. Ethyl gallate has a structure similar to that of GSS, but has three hydroxyl moieties and different physical properties, in which the pKa of GSS is higher than ethyl gallate which may be important in the phenol’s ability to modify lactate metabolism in SS.

The mechanism by which GSS caused a reduction of LA is not clear. One possibility is that since GSS had a greater effect on coupled respiration than did NE, this could make oxidative phosphorylation supernormal leading to enhanced hepatic clearance of lactate by this mechanism. However, against this possibility is the fact that maximal, uncoupled respiration did not increase in the gentisic septic group, a finding of which would also be expected to occur in this instance. Nevertheless, there are other mechanisms to consider. One possibility is that GSS could have decreased splanchnic production of lactate, or alternatively could have increased hepatic uptake of lactate by mechanisms that are independent of mitochondrial function. For instance, this could occur by enhancing hepatic gluconeogenesis, or by reducing glycolysis, since both of the latter mechanisms have been reported in other conditions. For instance, in asthma, β-agonist effects have been shown to produce elevations in lactate by enhancing glycolysis even under non-hypoxemic conditions [30]. On the other hand, in diabetes, metformin has been shown to suppress gluconeogenesis by inhibiting mitochondrial glycerophosphate dehydrogenase, which leads to enhanced lactate production in the cytosol [31]. The extent to which each of these mechanisms could play a role in GSS’s effect is not yet clear, but is worthy of future investigation.

In this study, we recognize moreover that in terms of the animal model used, there are a number of methodological issues that need to be addressed. We administered a high concentration of NE in the NE septic group, since, in the design of this study, we wanted to return MAP to the baseline value. We chose the latter high end-point to clearly demonstrate a change in MAP between the shock condition and the 5-h measurement with NE treatment. On the other hand, in clinical medicine, a much lower MAP end-point would be chosen with a corresponding lower concentration of NE. Whether a lower rate of NE infusion would provide a similar beneficial effect on hepatic mitochondrial function is not clear at this time.

Moreover, in the design of this study, although we attempted to keep Pwp close to the baseline value, there were slight changes in Pwp that occurred over the course of the study in the five groups. In the non-septic groups, we needed to constantly infuse intravenous volume to administer the anesthetic agents, so that Pwp rose slightly over the course of the study and there was net intravascular fluid retention. On the other hand, in the septic groups, particularly from the baseline to shock condition, there was a lot of third spacing (i.e., ascites was often found at autopsy) which tended to reduce Pwp. After the shock condition, however, when the vasoactive agents were added in the gentisic septic group and the NE septic group, there were increases in afterload which led to increases in Pwp. While intravenous fluids could easily be given when the Pwp was low, we did not want to phlebotomize that animal if the Pwp rose, because hemodynamic instability often follows this procedure [33]. Overall, however, the changes in Pwp were relatively small (see Table 3), and moreover, we do not think these changes would affect the hemodynamic findings to a significant extent. At a Pwp ~10 mmHg, the left ventricle is well-filled and the left ventricular diastolic volume pressure relationship is relatively flat with little changes in ventricular volume for a given change in Pwp [34]. This finding would limit any alterations in CO that would occur under the variations in Pwp found in the present study.

Finally, we noticed small changes in the blood chemistries in the non-septic control groups over the course of the experiment, particularly in creatine kinase which was probably related to some rhabdomyolysis because we positioned the animals in the supine position and did not turn the animal over the 10-h course of the study (see Additional file 3: Table S2, Additional file 4: Table S3, Additional file 5: Table S4, Additional file 6: Table S5 and Additional file 7: Table S6). To some extent, this may have produced skeletal muscle damage leading to mild renal dysfunction. However, the changes in blood chemistries in the non-septic groups were markedly less than those found in the septic groups, so that we do not think that these small changes in blood chemistries affected the major conclusions of the study.

## Conclusions

In a canine model of SS, we showed that, hepatic mitochondrial dysfunction evolved over the course of sepsis in which the predominant finding observed was a reduction in maximal OCR. Although NE was able to reverse hepatic mitochondrial dysfunction in SS, this effect was not associated with an improvement in LA. Thus, hepatic mitochondrial dysfunction is not a requirement for the development of LA in this SS model. In contrast, GSS had a greater effect than NE on reversing hepatic mitochondrial dysfunction and additionally caused a reduction in LA. As compared with NE, HR with GSS were lower, despite similar increases in MAP with the two treatments. Among other possibilities, we speculate that GSS could reduce LA by increasing hepatic uptake of lactate either by decreasing the rate of hepatic glycolysis or by increasing hepatic gluconeogenesis. Although the relationship between animal models and the human condition must be considered cautiously, we propose that GSS may be a novel therapy for the treatment for LA and cardiovascular collapse in SS.

## Abbreviations

AA, antimycin A; ADP, adenosine diphospate; ANOVA, analysis of variance; ATP, adenosine triphosphate; BSA, bovine serum albumin; CO, cardiac output; D5W, 5 % dextrose in water; DO_2_, oxygen delivery; EGTA, ethylene glycol tetraacetic acid; ETC., electron transport chain; FCCP, carbonyl cyanide p-triflouromethoxy-phenylhydrazone; GSS, gentisic acid sodium salt; H^+^, proton; Hb, hemoglobin; HEPES, 4-(2-hydroxyethyl)-1-piperazineethanesulfonic acid); HR, heart rates; LA, lactic acidosis; MAP, mean arterial pressure; MAS, mitochondrial assay solution; MD, myocardial depression; MSHE, mitochondrial isolation buffer; NADH, nicotinamide adenine dinucleotide; NE, norepinephrine; OCR, oxygen consumption rate; OH^−^, hydroxyl ion; pmf, proton motive force; Ppa, mean pulmonary artery pressure; Pwp, mean pulmonary wedge pressure; R, rotenone; Rap, mean right atrial pressure; ROS, reactive oxygen species; SNK, Student Newman Keuls multiple comparison test; SS, septic shock; SVR, systemic vascular resistance

## Additional files

Additional file 1:
**Figure S1.** In this figure, troponin, our index of myocardial injury, increased in the NE septic group, while it did not change in the other groups. GSS is gentisic acid sodium salt. Statistical analyses included two-way repeated measures analysis of variance and Student Newman Keuls (SNK) multiple comparison multiple comparison test. (DOC 170 kb) Additional file 2:
**Table S1.** Oxygen delivery in the five groups (ml/min/kg: mean ± SD). (DOC 30 kb) Additional file 3:
**Table S2.** Selective blood and hematology parameters in the non-septic control group (*n* = 16). (DOC 40 kb) Additional file 4:
**Table S3.** Selective blood and hematology parameters in the septic control group (*n* = 13). (DOC 40 kb) Additional file 5:
**Table S4.** Selective blood and hematology parameters in the gentisic septic group (*n* = 11). (DOC 40 kb) Additional file 6:
**Table S5.** Selective blood and hematology parameters in the norepinephrine septic group (*n* = 10). (DOC 39 kb) Additional file 7:
**Table 6.** Selective blood and hematology parameters in the gentisic acid sodium salt (GSS) non-septic group (*n* = 5). (DOC 39 kb)
