# Supplementary material for: Gentisic acid sodium salt, a phenolic compound, is superior to norepinephrine in reversing cardiovascular collapse, hepatic mitochondrial dysfunction and lactic acidemia in Pseudomonas aeruginosa septic shock in dogs
Source: Intensive Care Med Exp. 2016 Jul 26;4:24. doi: 10.1186/s40635-016-0095-0 (PMC4960072; doi:10.1186/s40635-016-0095-0)
Supplement: Additional file 1: — Figure S1. In this figure, troponin, our index of myocardial injury, increased in the NE septic group, while it did not change in the other groups. GSS is gentisic acid sodium salt. Statistical analyses included two-way repeated measures analysis of variance and Student Newman Keuls (SNK) multiple comparison multiple comparison test. (DOC 170 kb) [file 40635_2016_95_MOESM1_ESM.doc]

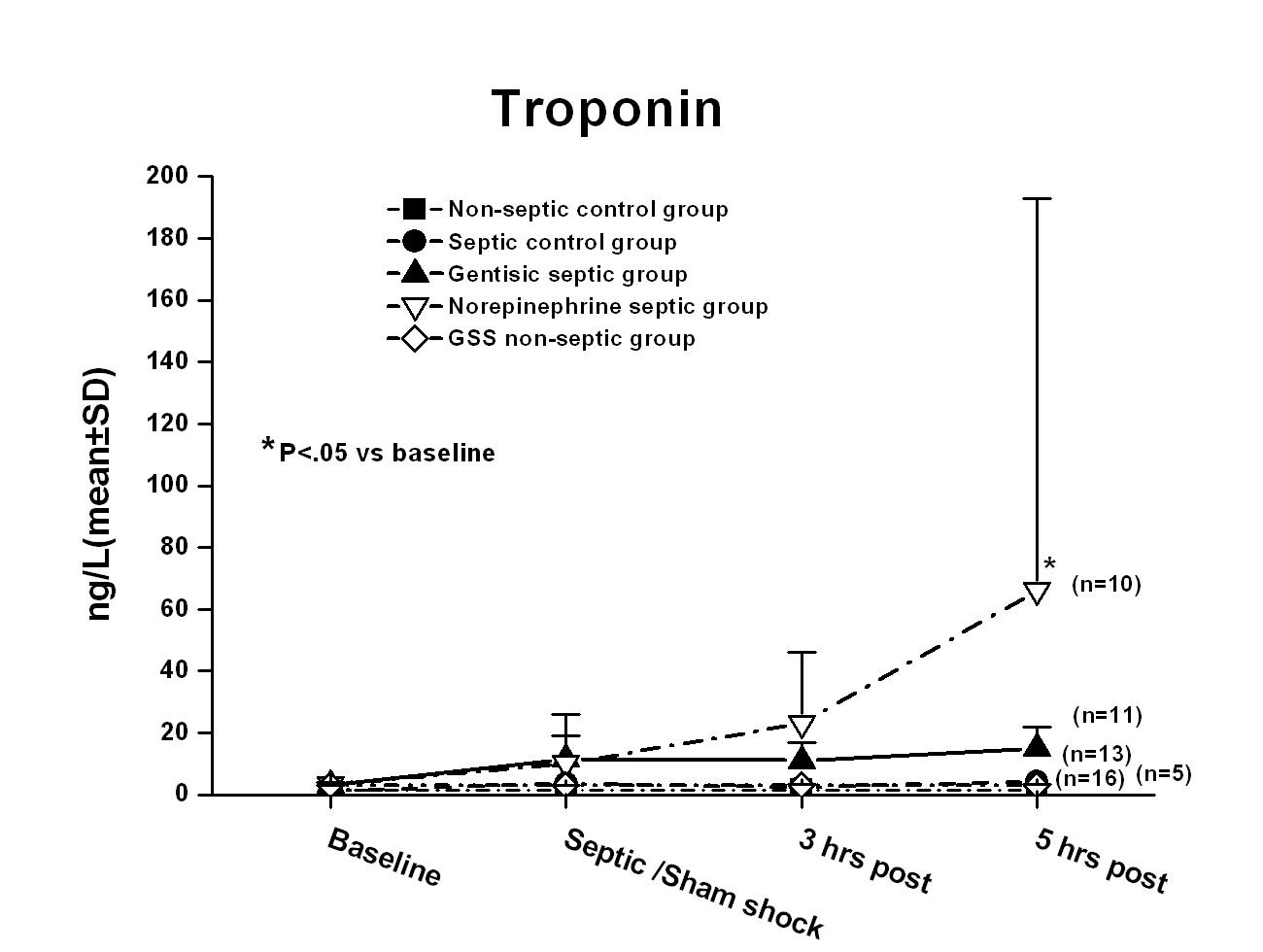


Legend to Additional file 1: Figure S1.

In this figure, troponin, our index of myocardial injury, increased in the NE septic group, while it did not change in the other groups. GSS is gentisic acid sodium salt. Statistical analyses included two-way repeated measures analysis of variance and Student Newman Keuls’ (SNK) multiple comparison multiple comparison test.
