# Supplementary material for: Gentisic acid sodium salt, a phenolic compound, is superior to norepinephrine in reversing cardiovascular collapse, hepatic mitochondrial dysfunction and lactic acidemia in Pseudomonas aeruginosa septic shock in dogs
Source: Intensive Care Med Exp. 2016 Jul 26;4:24. doi: 10.1186/s40635-016-0095-0 (PMC4960072; doi:10.1186/s40635-016-0095-0)
Supplement: Additional file 2: — Table S1. Oxygen delivery in the five groups (ml/min/kg: mean ± SD). (DOC 30 kb) [file 40635_2016_95_MOESM2_ESM.doc]

Additional file 2: Table S1. Oxygen delivery in the 5 groups (ml/min/kg: mean ±SD)

|  | **Baseline** | **Septic/sham shock/condition** | **3 hrs post** | **5 hrs post** |
| --- | --- | --- | --- | --- |
| **Non-septic control group (n=16)** | 25 ±7 | 25 ±9 | 22 ±6 | 20 ±4 |
| **Septic control group (n=13)** | 22 ±7 | 24 ±8 | 21 ±9 | 17±9 |
| **Gentisic septic group (n=11)** | 18 ±3 | 26 ±6 | 20 ±7 | 20 ±9 |
| **Norepinephrine septic group (n=10)** | 20±3 | 26±.11 | 29 ±8 | 28±10+ |
| **Gentisic non-septic group (n=5)** | 20±4 | 24 ±3 | 28 ±7 | 27 ±10 |

Statistics by two way analysis of variance (ANOVA) and Student Newman Keuls’ multiple comparison test that included the 5 groups and four time periods. There was a s significant increase (+P <.05) vs septic control group and gentisic septic group
