# Supplementary material for: Gentisic acid sodium salt, a phenolic compound, is superior to norepinephrine in reversing cardiovascular collapse, hepatic mitochondrial dysfunction and lactic acidemia in Pseudomonas aeruginosa septic shock in dogs
Source: Intensive Care Med Exp. 2016 Jul 26;4:24. doi: 10.1186/s40635-016-0095-0 (PMC4960072; doi:10.1186/s40635-016-0095-0)
Supplement: Additional file 3: — Table S2. Selective blood and hematology parameters in the non-septic control group (n = 16). (DOC 40 kb) [file 40635_2016_95_MOESM3_ESM.doc]

Additional file 3: Table S2. Selective blood and hematology parameters in the non- septic control group (n=16)

|  | **Baseline** | **Sham shock** | **3hrs posttreatment** | **5 hrs posttreatment** |
| --- | --- | --- | --- | --- |
| **Hemoglobin (grams/L)** | 124±16 | 130±22 | 120±17 | 118±21 |
| **WBC (10^9^/L)** | 3.2±1.2@ | 5.2.1±3.9*^#@!$^ | 7.43±3.9*^#@!$^ | 8.2±.3.3*^#@!$^ |
| **AST (IU)** | 20±7 | 41±26^#!^ | 53±28^#@!^ | 71±39#@!^ |
| **ALT (IU)** | 32±15 | 49±31^#@!^ | 61±39^#@!^ | 69±48^#@!^ |
| **LD (IU)** | 42±16 | 74±19^#@^ | 71±21^#@!^ | 86±32^#@!^ |
| **CK (IU)** | 98±27 | 214±220 | 768±830^!^ | 1588±1596^!$^ |
| **Creatinine clearance (ml/min)** | 114±.62 | 68±.23 | 54±11* | 62±30*^@#!^ |
| **Arterial pH** | 7.41±.05 | 7.37±.05^#@!^ | 7.36±.06^#@!^ | 7.35±0.04*^#@!^ |
| **Mixed venous PO_2_** | 51±7 | 56±26 | 50±7 | 49±9 |

Mean (± SD). Measurements were obtained at baseline, at the sham shock condition, and after 3 hrs and 5 hrs post pacebo treatment. ALT, AST, LD, CK (in international units) are alanine transaminase, aspartate transaminase, lactate dehydrogenase, and creatine kinase respectively. *P<.05 vs baseline; ^#^P<.05 vs septic control group; @P<.05 vs gentisic septic group; ^!^P<.05 vs norepinephrine septic group; ^$^P<.05 vs gentisic acid sodium salt non-septic group; by two way analysis of variance and Student Newman Keuls’ multiple comparison test.

**Table 2.** **Selective blood chemistries and hematology parameters in the early *treatment protocol***
