# Supplementary material for: Gentisic acid sodium salt, a phenolic compound, is superior to norepinephrine in reversing cardiovascular collapse, hepatic mitochondrial dysfunction and lactic acidemia in Pseudomonas aeruginosa septic shock in dogs
Source: Intensive Care Med Exp. 2016 Jul 26;4:24. doi: 10.1186/s40635-016-0095-0 (PMC4960072; doi:10.1186/s40635-016-0095-0)
Supplement: Additional file 4: — Table S3. Selective blood and hematology parameters in the septic control group (n = 13). (DOC 40 kb) [file 40635_2016_95_MOESM4_ESM.doc]

Additional file 4: Table S3. Selective blood and hematology parameters in the septic control group (n=13)

|  | **Baseline** | **Septic shock** | **3hrs posttreatment** | **5 hrs posttreatment** |
| --- | --- | --- | --- | --- |
| **Hemoglobin (grams/L)** | 122±14 | 107±36 | 113±39 | 109±35 |
| **WBC (10^9^/L)** | 3.6±1.8 | 1.1±.6*^+$^ | 1.3±.9*^+@!$^ | 0.9±.4*^+!$^ |
| **AST (IU)** | 38±54 | 159±79*^+^ | 253±126*^+$^ | 380±294*^+$^ |
| **ALT (IU)** | 59±71 | 155±90^+^ | 254±149*^+$^ | 334±202*^+@$^ |
| **LD (IU)** | 44±23 | 164±72*^+^ | 259±84*^+$^ | 375±231*^+$^ |
| **CK (IU)** | 219±373 | 406±328 | 1317±2027^!^ | 1890±2545^!^ |
| **Creatinine clearance (ml/min)** | 99±.42 | 52±.33 | 22±14* | 15±14* |
| **Arterial pH** | 7.39±.05 | 7.27±.06*^+!$^ | 7.21±.08*^+$^ | 7.13±0.12*^+@$^ |
| **Mixed venous PO_2_** | 50±7 | 53±10 | 53±12 | 52±10 |

Mean (± SD). Measurements were obtained at baseline, at the septic shock condition, and after 3 hrs and 5 hrs post placebo treatment. ALT, AST, LD, CK (in international units) are alanine transaminase, aspartate transaminase, lactate dehydrogenase, and creatine kinase respectively. *P<.05 vs baseline; ^+^P<.05 vs non-septic control group; @P<.05 vs gentisic septic group; ^!^P<.05 vs norepinephrine septic group; ^$^P<.05 vs gentisic acid sodium salt non-septic group; by two way analysis of variance and Student Newman Keuls’ multiple comparison test.

**Table 2.** **Selective blood chemistries and hematology parameters in the early *treatment protocol***
