# Supplementary material for: Gentisic acid sodium salt, a phenolic compound, is superior to norepinephrine in reversing cardiovascular collapse, hepatic mitochondrial dysfunction and lactic acidemia in Pseudomonas aeruginosa septic shock in dogs
Source: Intensive Care Med Exp. 2016 Jul 26;4:24. doi: 10.1186/s40635-016-0095-0 (PMC4960072; doi:10.1186/s40635-016-0095-0)
Supplement: Additional file 5: — Table S4. Selective blood and hematology parameters in the gentisic septic group (n = 11). (DOC 40 kb) [file 40635_2016_95_MOESM5_ESM.doc]

Additional file 5: Table S4. Selective blood and hematology parameters in the gentisic septic group (n=11)

|  | **Baseline** | **Septic shock** | **3hrs posttreatment** | **5 hrs posttreatment** |
| --- | --- | --- | --- | --- |
| **Hemoglobin (grams/L)** | 112±13 | 108±34 | 125±28 | 130±22 |
| **WBC (10^9^/L)** | 2.4±1.4^$^ | 1.1±0.9^+$^ | 1.5±1.2^+$^ | 1.81±1.8^+$^ |
| **AST (IU)** | 29±31 | 143±113 | 235±167*^+$^ | 279±208*^+!$^ |
| **ALT (IU)** | 51±29 | 161±132 | 209±158*^+!^ | 225±171*^+#^ |
| **LD (IU)** | 36±17 | 150±101* | 267±125*^+$^ | 333±139*^+$^ |
| **CK (IU)** | 117±106 | 521±750 | 1847±1763^!^ | 2857±2527*^!^ |
| **Creatinine clearance (ml/min)** | 78±39 | 46±42 | 29±18 | 14±14* |
| **Arterial pH** | 7.38±.03 | 7.26±.05*^+$^ | 7.24±.08*^+$^ | 7.20±0.06*^+#$^ |
| **Mixed venous PO_2_** | 45±6 | 56±7 | 50±6 | 48±7 |

Mean (± SD). Measurements were obtained at baseline, at the septic shock condition, and after 3 hrs and 5 hrs post gentisic acid sodium salt. ALT, AST, LD, CK (in international units) are alanine transaminase, aspartate transaminase, lactate dehydrogenase, and creatine kinase respectively. *P<.05 vs baseline; ^+^P<.05 vs non-septic control group; ^#^P<.05 vs septic control group; ^!^P<.05 vs norepinephrine septic group; ^$^P<.05 vs gentisic acid sodium salt non-septic group; by two way analysis of variance and Student Newman Keuls’ multiple comparison test.

**Table 2.** **Selective blood chemistries and hematology parameters in the early *treatment protocol***
