# Supplementary material for: Gentisic acid sodium salt, a phenolic compound, is superior to norepinephrine in reversing cardiovascular collapse, hepatic mitochondrial dysfunction and lactic acidemia in Pseudomonas aeruginosa septic shock in dogs
Source: Intensive Care Med Exp. 2016 Jul 26;4:24. doi: 10.1186/s40635-016-0095-0 (PMC4960072; doi:10.1186/s40635-016-0095-0)
Supplement: Additional file 6: — Table S5. Selective blood and hematology parameters in the norepinephrine septic group (n = 10). (DOC 39 kb) [file 40635_2016_95_MOESM6_ESM.doc]

Additional file 6: Table S5. Selective blood and hematology parameters in the norepinephrine septic group (n=10)

|  | **Baseline** | **Septic shock** | **3hrs posttreatment** | **5 hrs posttreatment** |
| --- | --- | --- | --- | --- |
| **Hemoglobin (grams/L)** | 124±15 | 124±43 | 151±13*^+#@^ | 152±21*^+#@^ |
| **WBC (10^9^/L)** | 3.6±1.7 | 1.4±1.0*^+$^ | 2.5±1.8^+$^ | 3.1±2.3^+$#^ |
| **AST (IU)** | 21±6 | 187±118* | 344±191*^+$^ | 407±178*^+@$^ |
| **ALT (IU)** | 30±8 | 179±157*^+^ | 305±272*^+@$^ | 307±208*^+$^ |
| **LD (IU)** | 43±23 | 177±97*^+^ | 315±113*^+$^ | 409±167*^+$^ |
| **CK (IU)** | 106±47 | 1557±3174 | 4573±5173*^+#@^ | 6273±5789*^+#@^ |
| **Creatinine clearance (ml/min)** | 127±117 | 41±.18* | 30±17* | 24±22* |
| **Arterial pH** | 7.38±.05 | 7.26±.04*^+$^ | 7.20±.04*^+$^ | 7.16±0.06*^+$^ |
| **Mixed venous PO_2_** | 51±6 | 52±4 | 58±8 | 58±7 |

Mean (± SD). Measurements were obtained at baseline, at the septic shock condition, and after 3 hrs and 5 hrs post norepinephrine treatment. ALT, AST, LD, CK (in international units) are alanine transaminase, aspartate transaminase, lactate dehydrogenase, and creatine kinase respectively. *P<.05 vs baseline; ^+^P<.05 vs non-septic control group; ^#^P<.05 vs septic control group; @P<.05 vs gentisic septic group; ^$^P<.05 vs gentisic acid sodium salt non-septic group; by two way analysis of variance and Student Newman Keuls’ multiple comparison test.

**Table 2.** **Selective blood**
