# Supplementary material for: Gentisic acid sodium salt, a phenolic compound, is superior to norepinephrine in reversing cardiovascular collapse, hepatic mitochondrial dysfunction and lactic acidemia in Pseudomonas aeruginosa septic shock in dogs
Source: Intensive Care Med Exp. 2016 Jul 26;4:24. doi: 10.1186/s40635-016-0095-0 (PMC4960072; doi:10.1186/s40635-016-0095-0)
Supplement: Additional file 7: — Table 6. Selective blood and hematology parameters in the gentisic acid sodium salt (GSS) non-septic group (n = 5). (DOC 39 kb) [file 40635_2016_95_MOESM7_ESM.doc]

Additional file 8: Table S6. Selective blood and hematology parameters in the gentisic acid sodium salt (GSS) non-septic group (n=5)

|  | **Baseline** | **Sham shock** | **3hrs posttreatment** | **5 hrs posttreatment** |
| --- | --- | --- | --- | --- |
| **Hemoglobin (grams/L)** | 126±8 | 136±11 | 129±6 | 127±11 |
| **WBC (10^9^/L)** | 5.4±1.3 | 14±3*^+#@!^ | 12±2.3*^+#@!^ | 15±2.7*^+#@!^ |
| **AST (IU)** | 24±8 | 41±16 | 75±27^#@^ | 113±25^#@!^ |
| **ALT (IU)** | 74±71 | 90±52 | 92±48^#!^ | 96±50^#!^ |
| **LD (IU)** | 36±14 | 59±16 | 91±26^#@!^ | 135±30^#@!^ |
| **CK (IU)** | 114±32 | 463±173 | 2284±837 | 4297±1218*^+^ |
| **Creatinine clearance (ml/min)** | 115±46 | 97±52@ | 75±21# | 65±46 |
| **Arterial pH** | 7.42±.07 | 7.38±.09^#@!^ | 7.35±.04^#@!^ | 7.32±0.03^#@!^ |
| **Mixed venous PO_2_** | 45±3 | 49±7 | 51±3 | 54±9 |

Mean (± SD). Measurements were obtained at baseline, at the sham shock condition, and after 3 hrs and 5 hrs post GSS. ALT, AST, LD, CK (in international units) are alanine transaminase, aspartate transaminase, lactate dehydrogenase, and creatine kinase respectively. *P<.05 vs baseline; ^+^P<.05 vs non-septic control group; ^#^P<.05 vs septic control group; !P<.05 vs norepinephrine septic group; @P<.05 vs gentisic septic group; by two way analysis of variance and Student Newman Keuls’ multiple comparison test.

**Table 2.** **Selective blood chemistries and hematology parameters in the early *treatment protocol***
